# Buffalo Academy of Medicine

**Published:** 1899-01

**Authors:** Thomas F. Dwyer


### Society Proceedings.

### BUFFALO ACADEMY OF MEDICINE.

Reported by THOMAS F. DWYER, M. D., Secretary.

THE regular quarterly meeting was held at the Academy parlors, Tuesday evening, December 6, 1898.

The President, Dr. ROSWELL PARK, called the meeting to order at 9 o'clock p. m.

The minutes of the last quarterly meeting were read and approved.

The council recommended the election for resident membership of the following applicants: Drs. Prescott Le Breton, Wm. R. Henderson, Lewis H. Denton, James A. Gibson and L. E. McC. Pomery. They were separately balloted for and elected Fellows of the Academy.

The resignation of the treasurer, Dr. Charles S. Jewett, who has gone to Europe, was read and accepted.

Dr. Eugene A. Smith was nominated and elected to that office for the unexpired term.

The section of surgery provided the following program: The curability of cancer, N. Jacobson, M.D., professor of clinical surgery, University of Syracuse. Discussion by Drs. Parmenter, Gaylord, Pease, Park, Blaauw, Benedict and Van Peyma. Colles's fracture, by Edward M. Dooley, M. D. Discussion by Drs. Jacobson, Grosvenor, Walsh and Van Peyma.

On motion the thanks of the Academy were tendered Dr. Jacobson for coming to Buffalo to read his interesting paper.

Attendance, 49. Adjourned at 11 p. m.

